# Supplementary material for: Total Synthesis of Eliglustat via Diastereoselective Amination of Chiral para-Methoxycinnamyl Benzyl Ether
Source: Molecules. 2022 Apr 18;27(8):2603. doi: 10.3390/molecules27082603 (PMC9029353; doi:10.3390/molecules27082603)
Supplement: Supplementary file 1 [file molecules-27-02603-s001.zip › molecules-1669532-supplementary.pdf]

molecules

Article

Total Synthesis of Eliglustat via Diastereoselective Amination of Chiral *para*-Methoxycinnamyl Benzyl Ether

Younggyu Kong, Pulla Reddy Boggu, Gi Min Park, Yeon Su Kim, Seong Hwan An, In Su Kim and Young Hoon Jung *

School of Pharmacy, Sungkyunkwan University, Suwon 16419, Korea
* Correspondence: yhjung@skku.edu; Tel.: +82-31-290-7711; fax: +82-31-292-8800

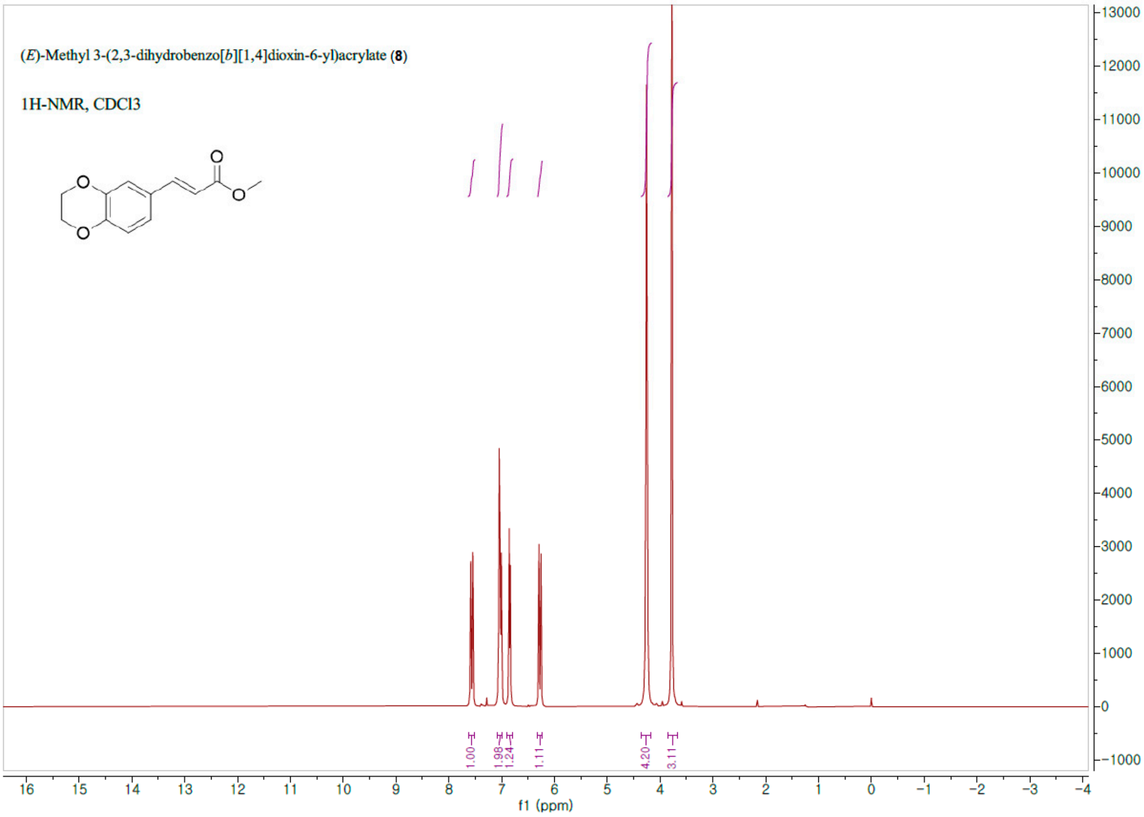

Figure S1. H and 13C NMR Spectra-S2-S11.

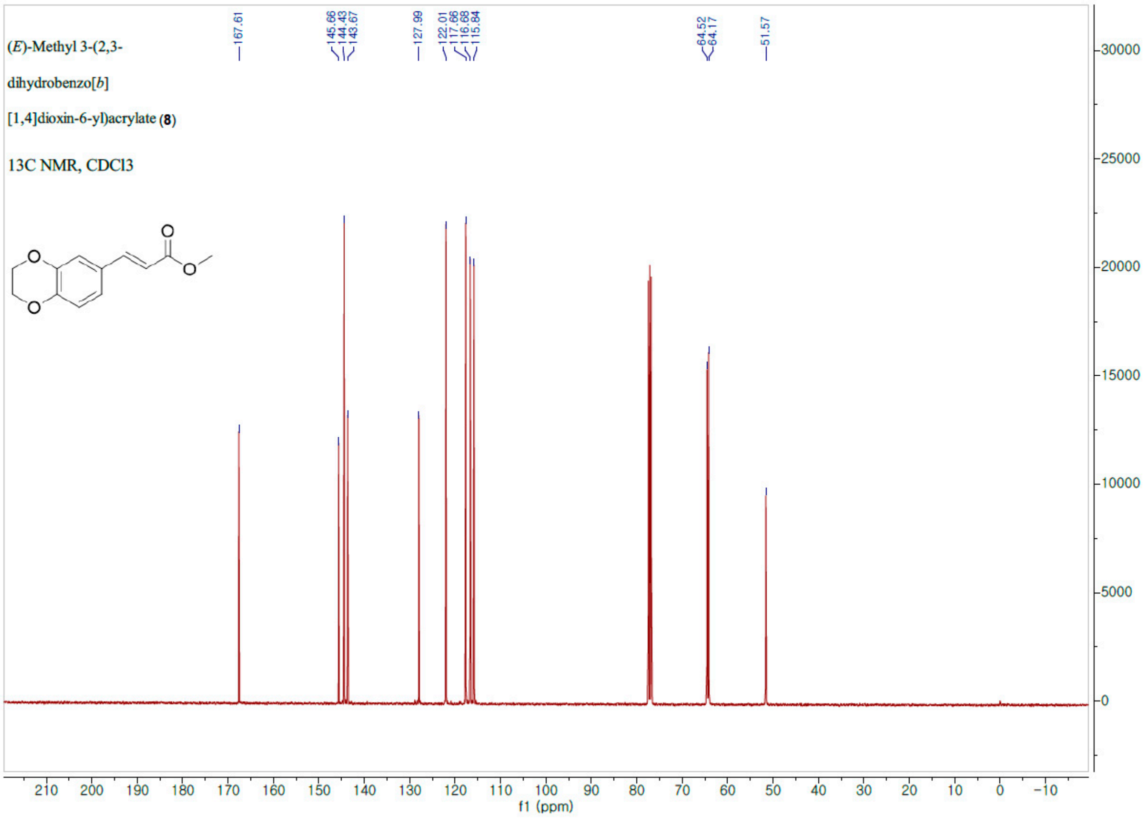

Figure S2. H and 13C NMR Spectra-S2-S11.

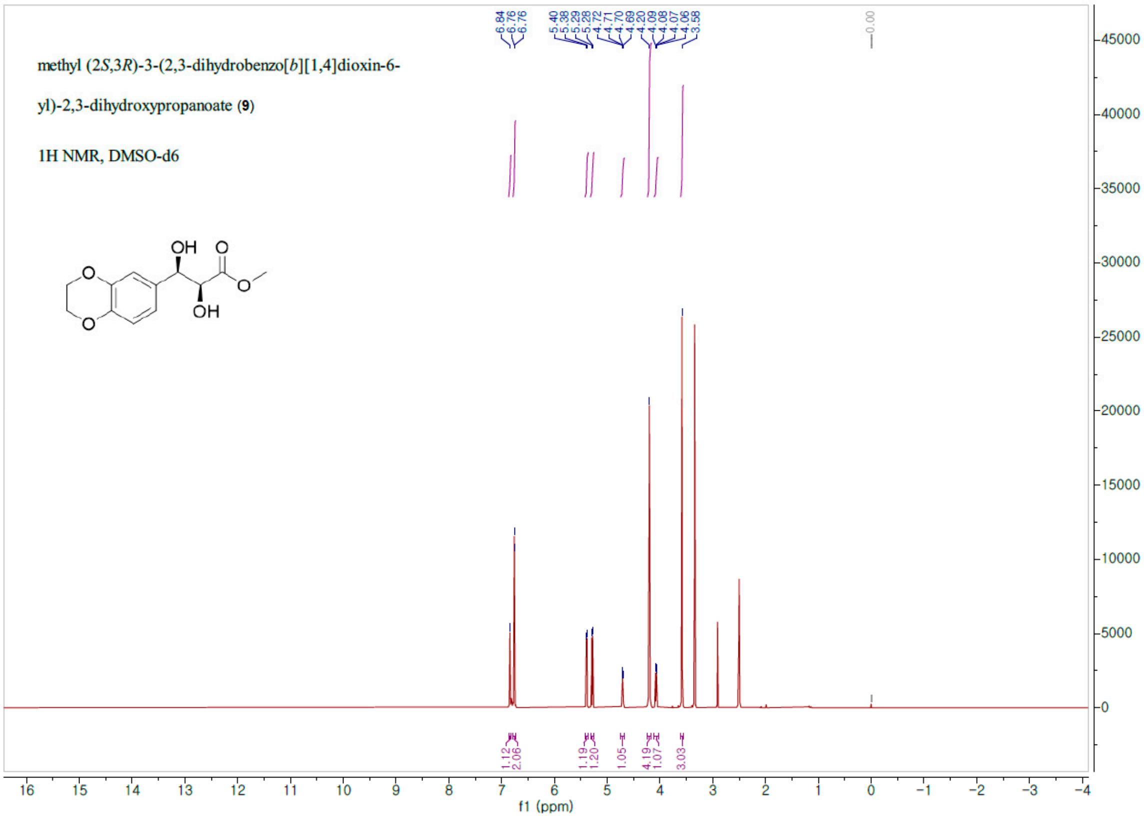

Figure S3. H and 13C NMR Spectra-S2-S11.

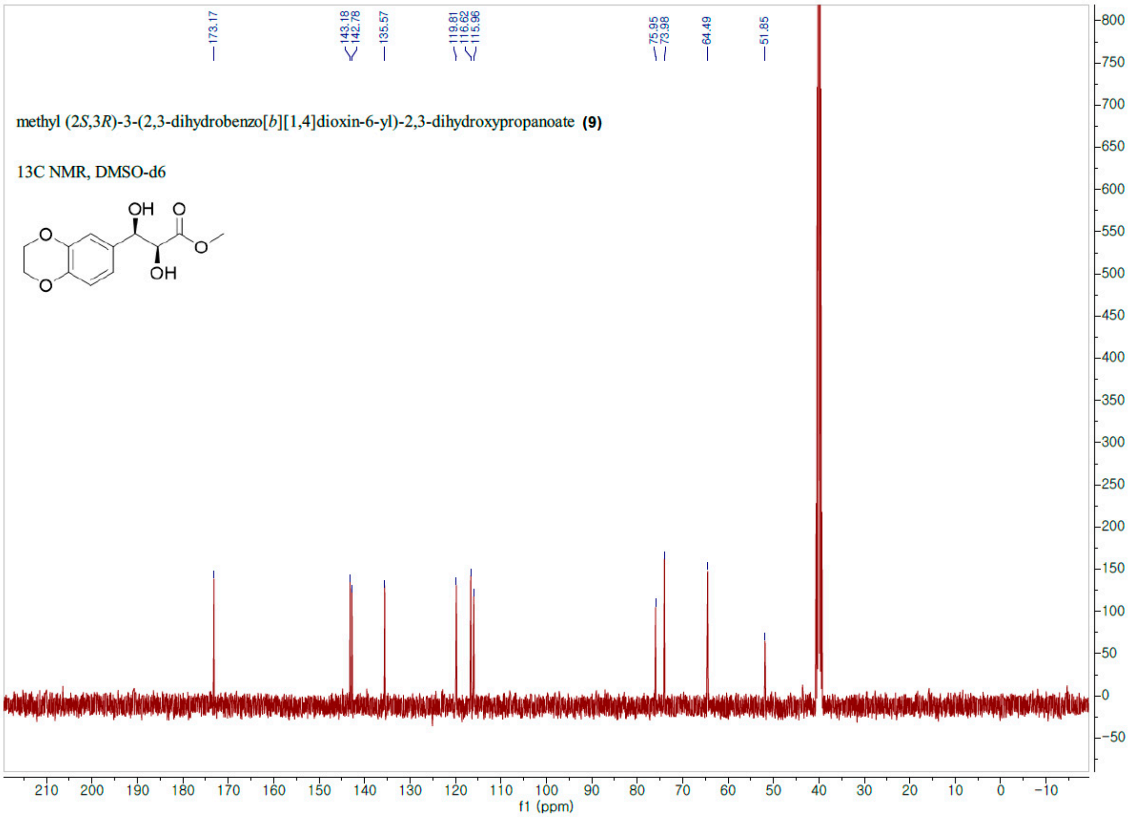

Figure S4. H and 13C NMR Spectra-S2-S11.

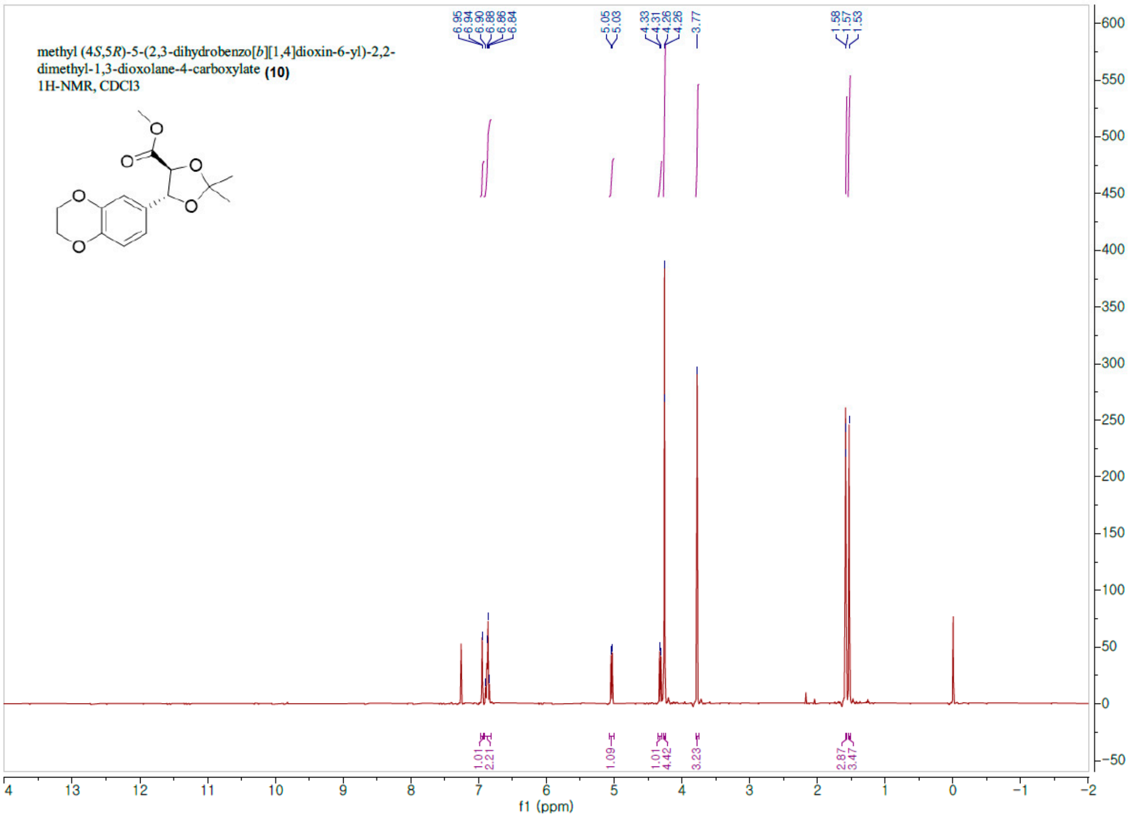

Figure S5. H and 13C NMR Spectra-S2-S11.

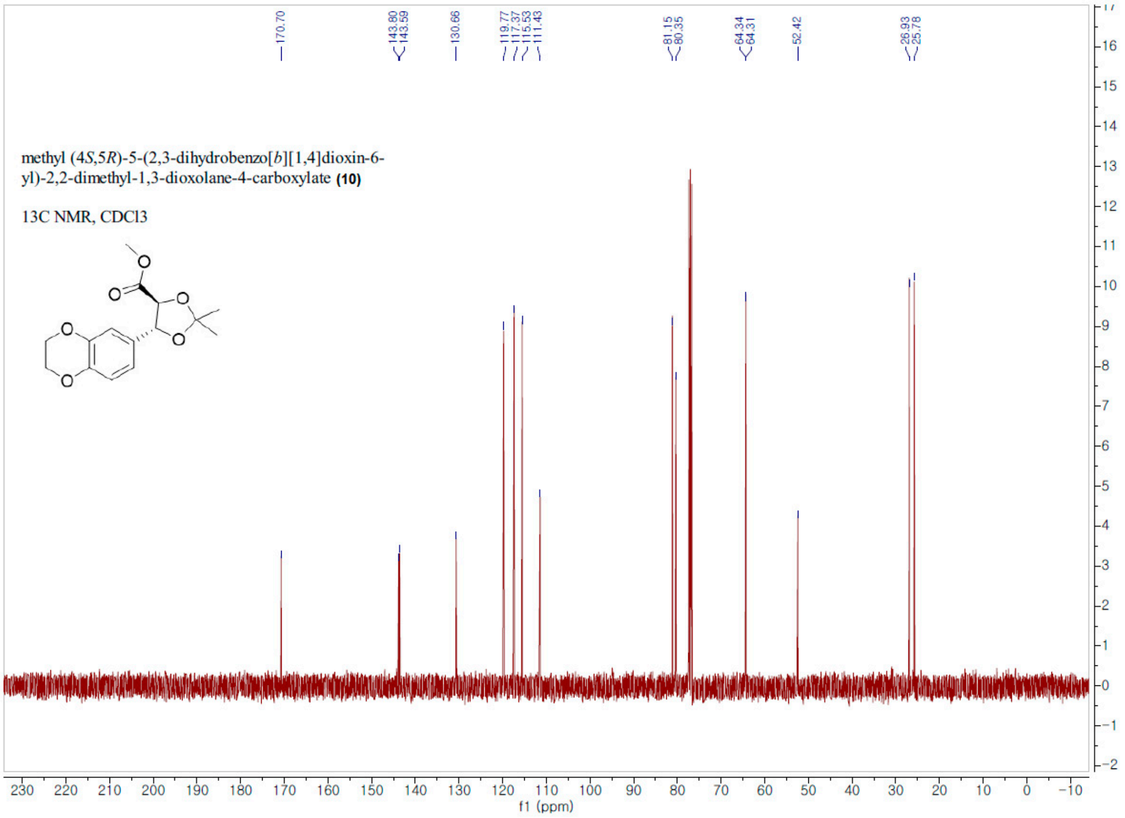

Figure S6. H and 13C NMR Spectra-S2-S11.

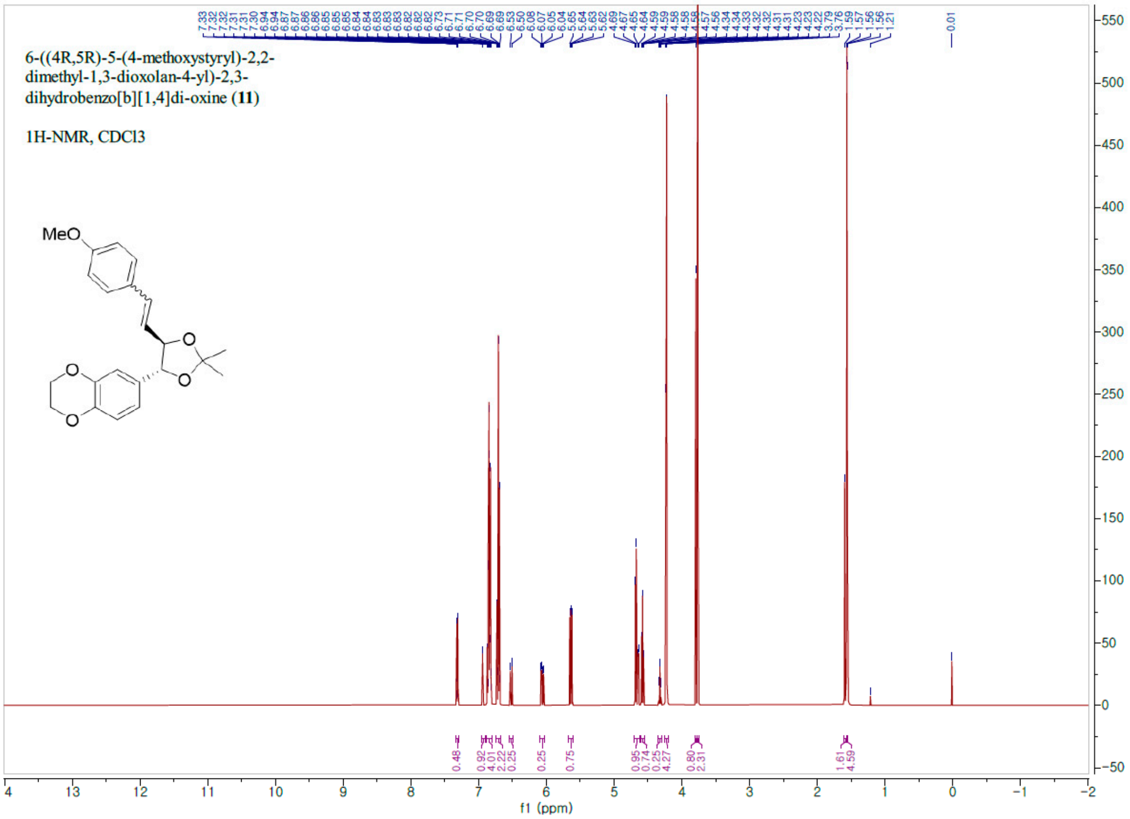

Figure S7. H and 13C NMR Spectra-S2-S11.

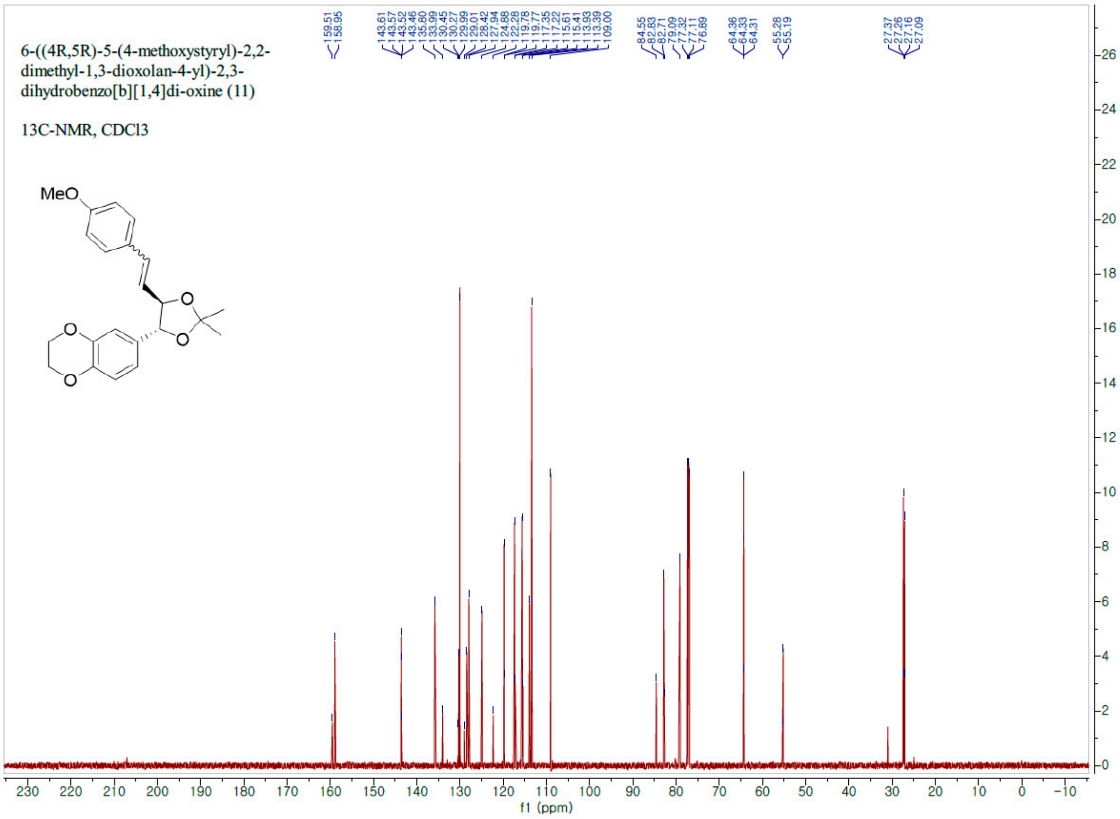

Figure S8. H and 13C NMR Spectra-S2-S11.

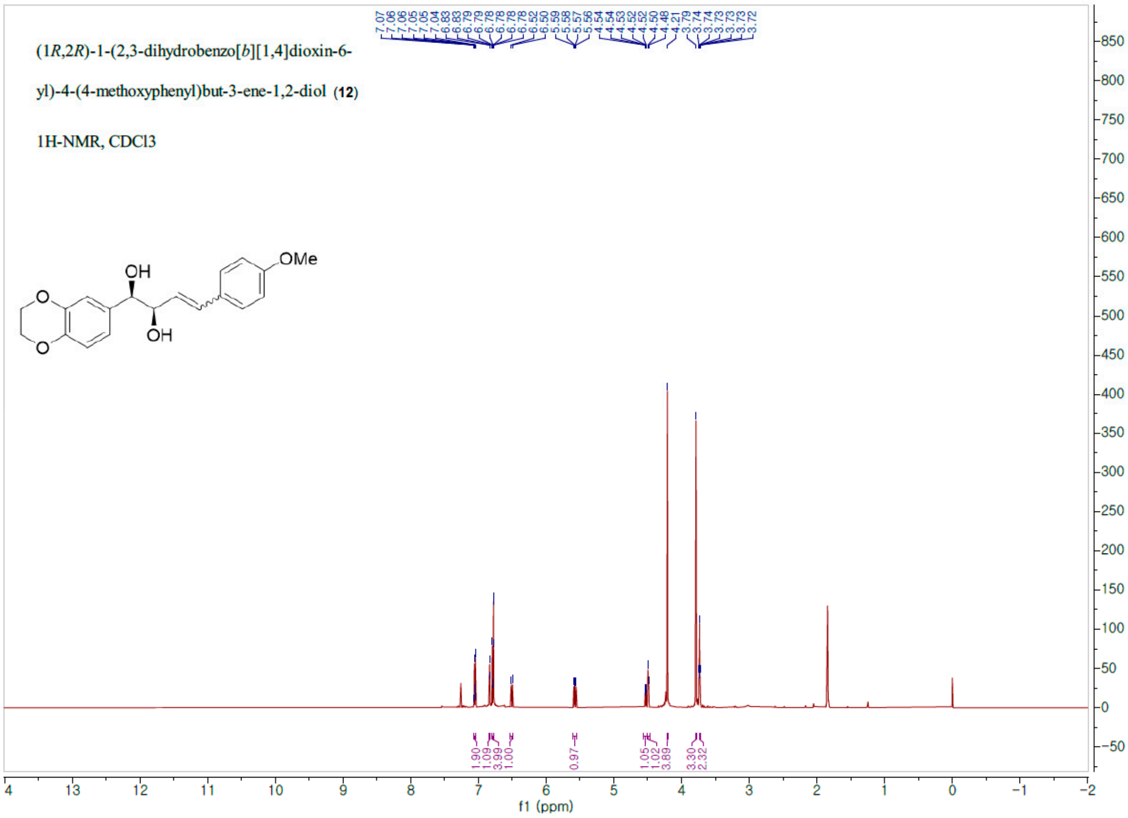

Figure S9. H and 13C NMR Spectra-S2-S11.

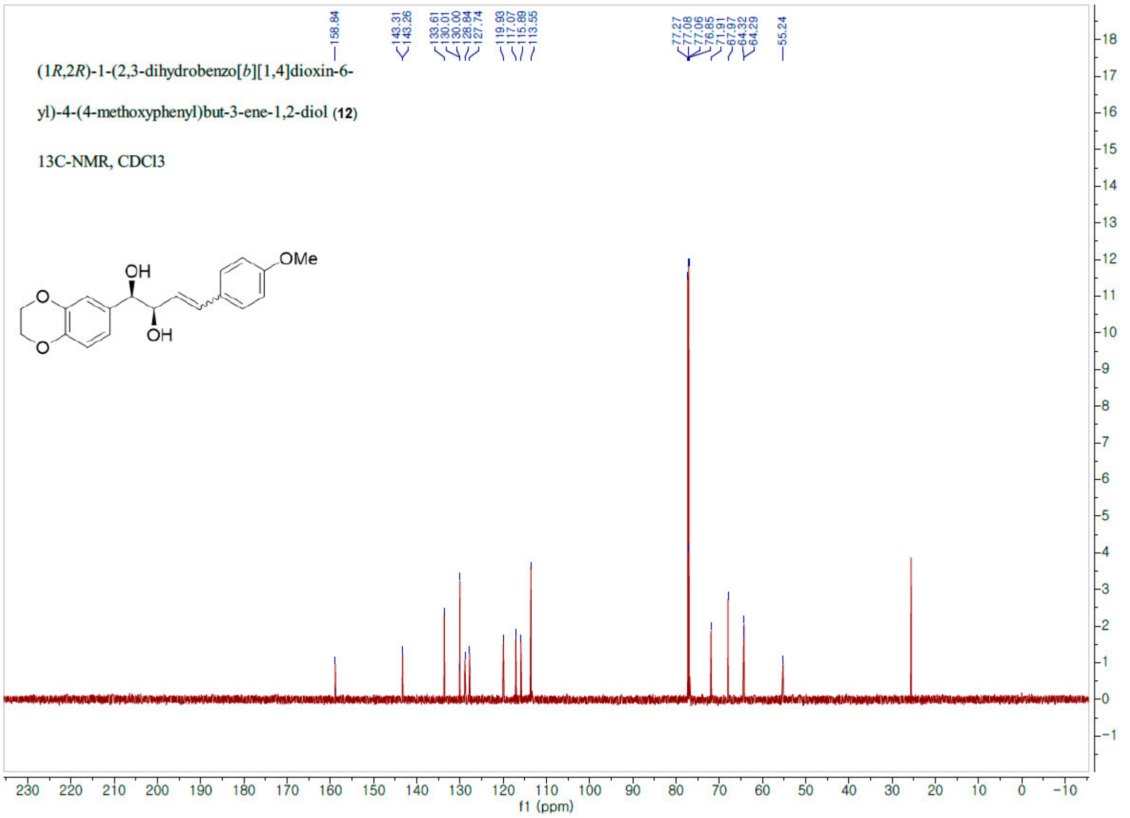

Figure S10. H and 13C NMR Spectra-S2-S11.

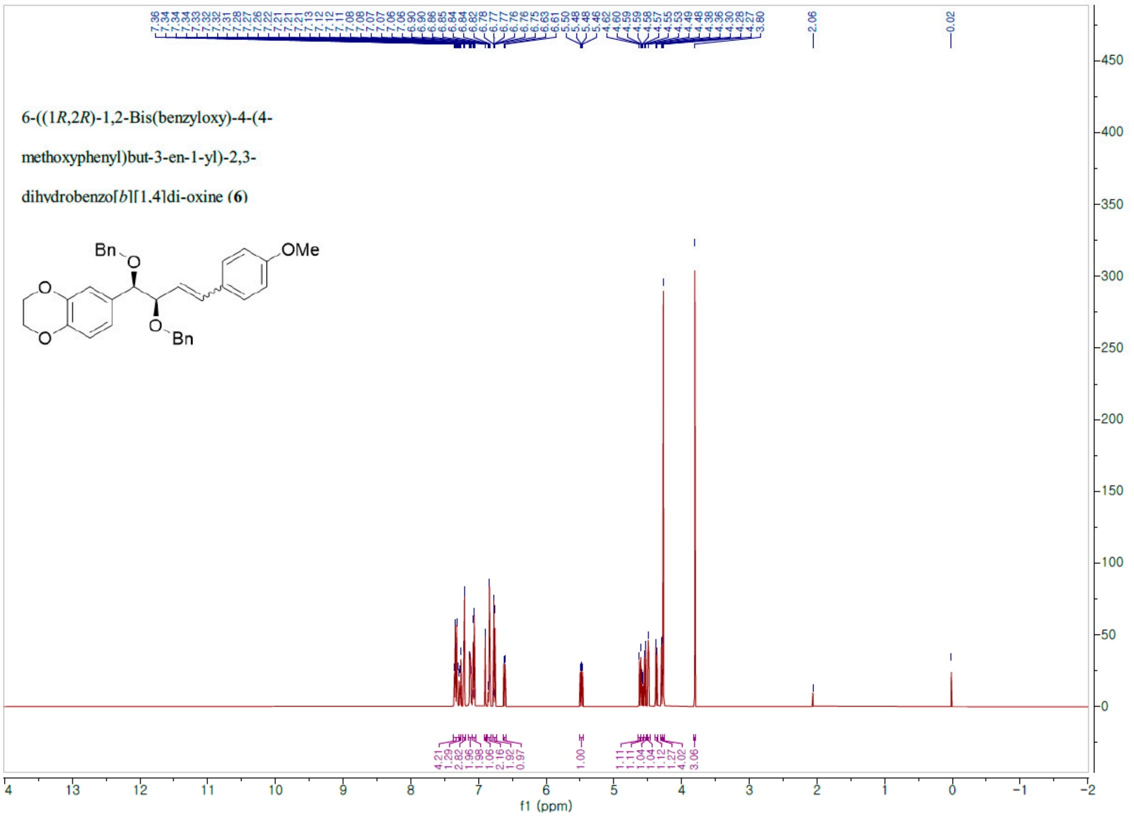

Figure S11. H and 13C NMR Spectra-S2-S11.

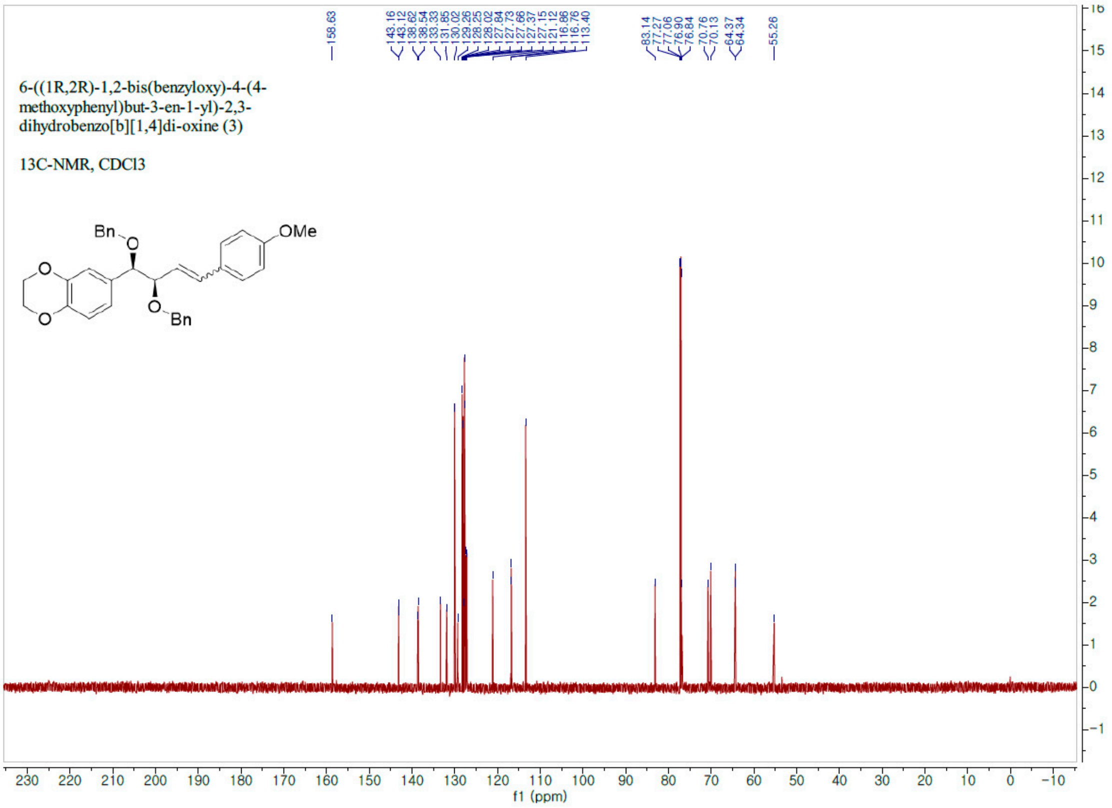

Figure S12. H and 13C NMR Spectra-S2-S11.

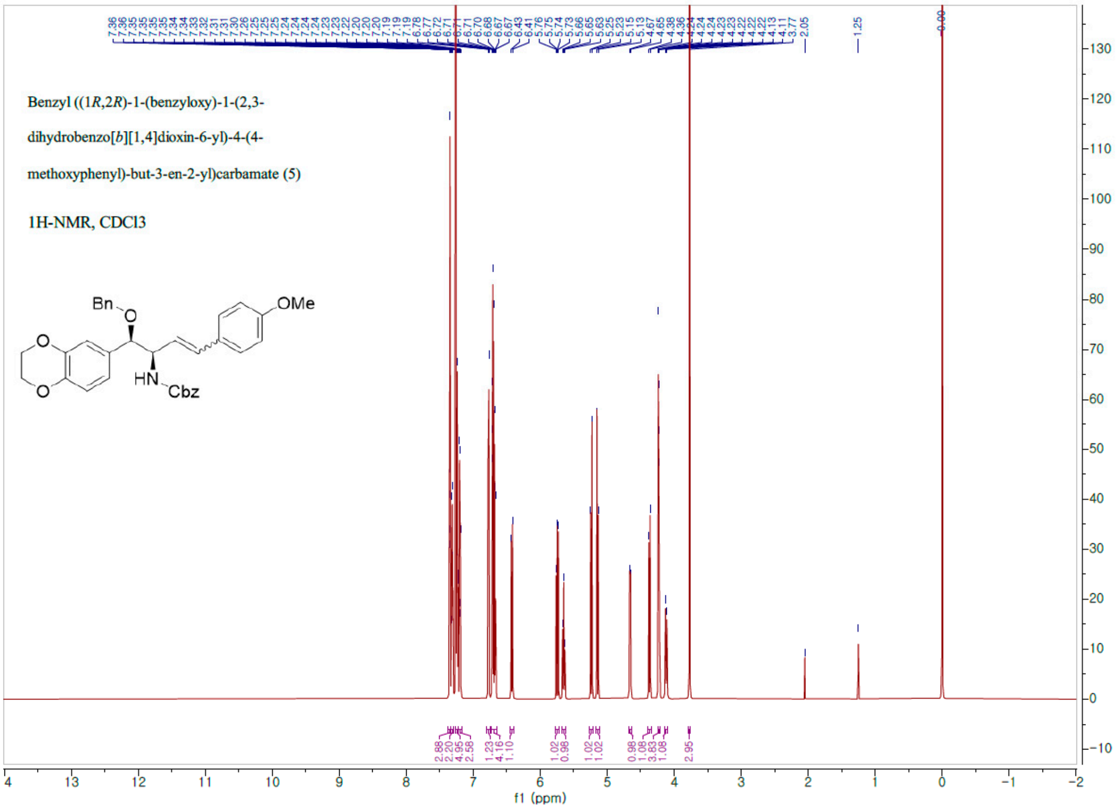

Figure S13. H and 13C NMR Spectra-S2-S11.

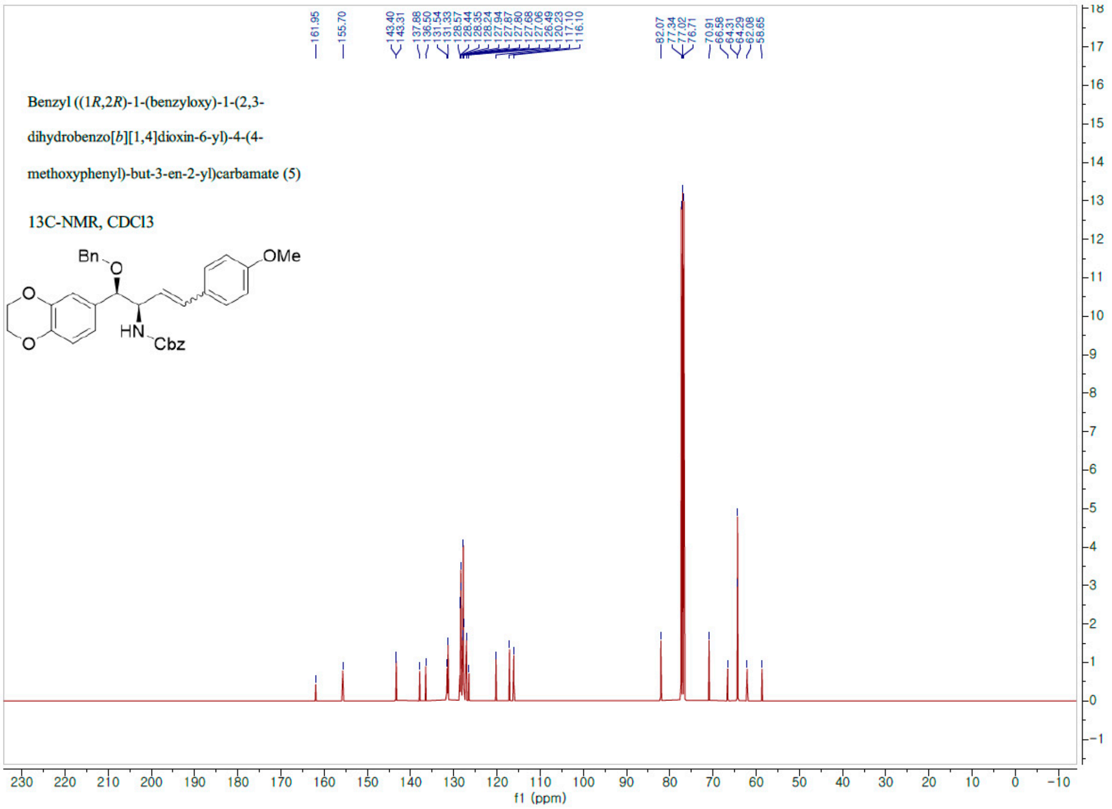

Figure S14. H and 13C NMR Spectra-S2-S11.

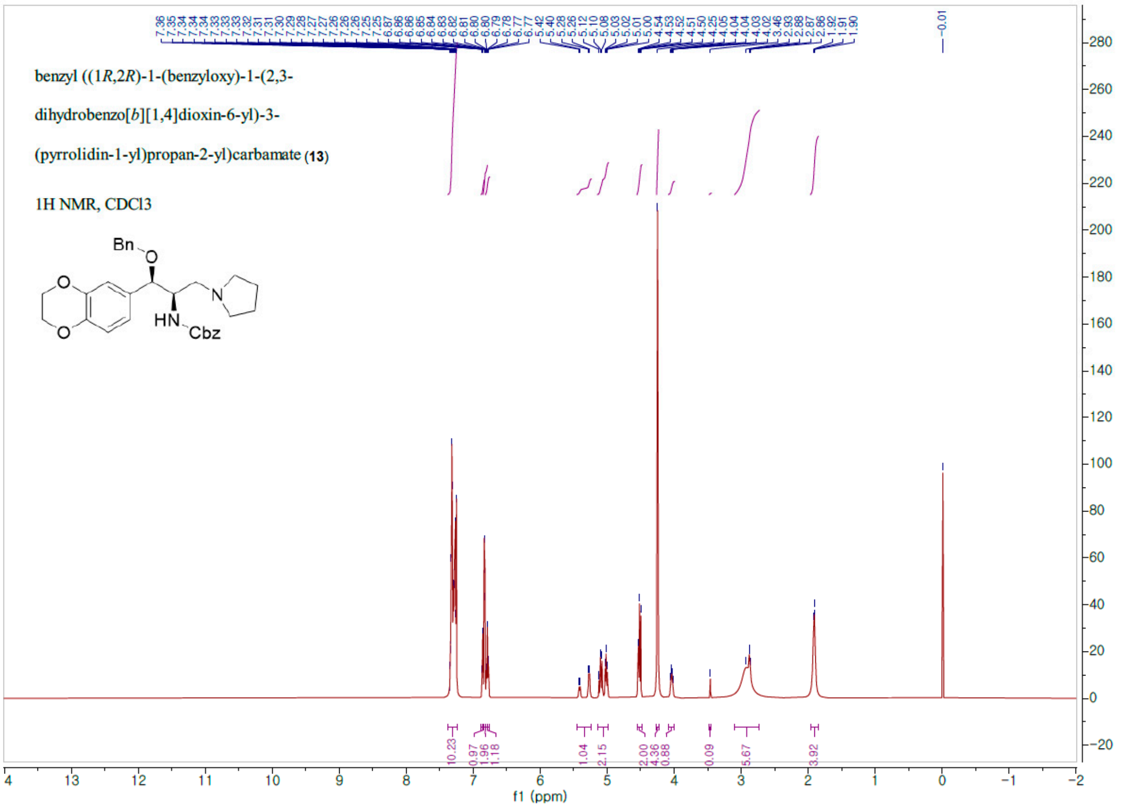

Figure S15. H and 13C NMR Spectra-S2-S11.

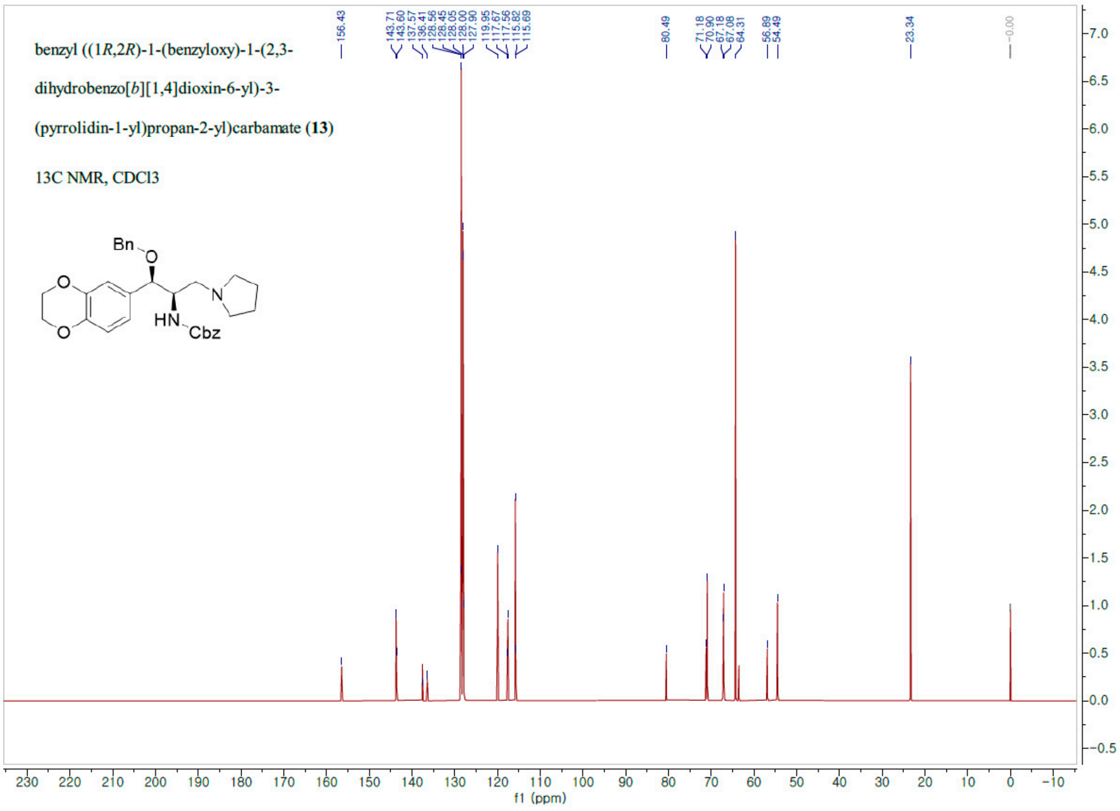

Figure S16. H and 13C NMR Spectra-S2-S11.

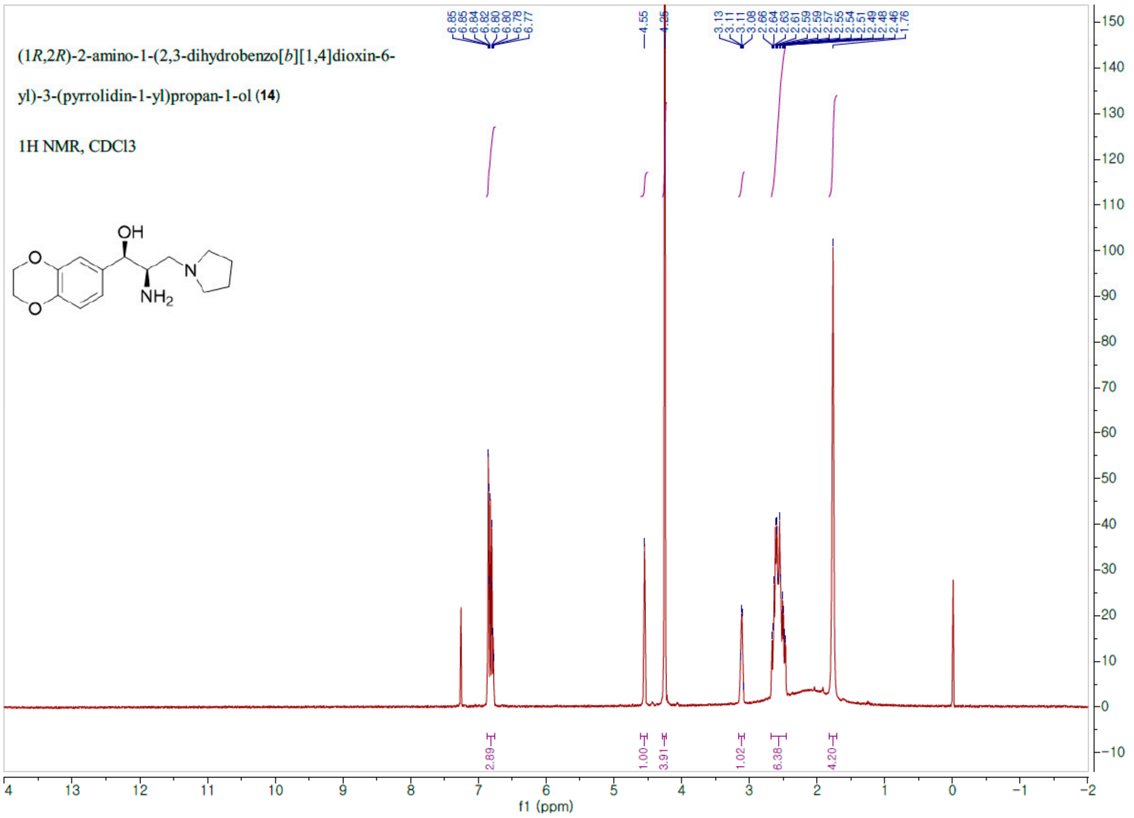

Figure S17. H and 13C NMR Spectra-S2-S11.

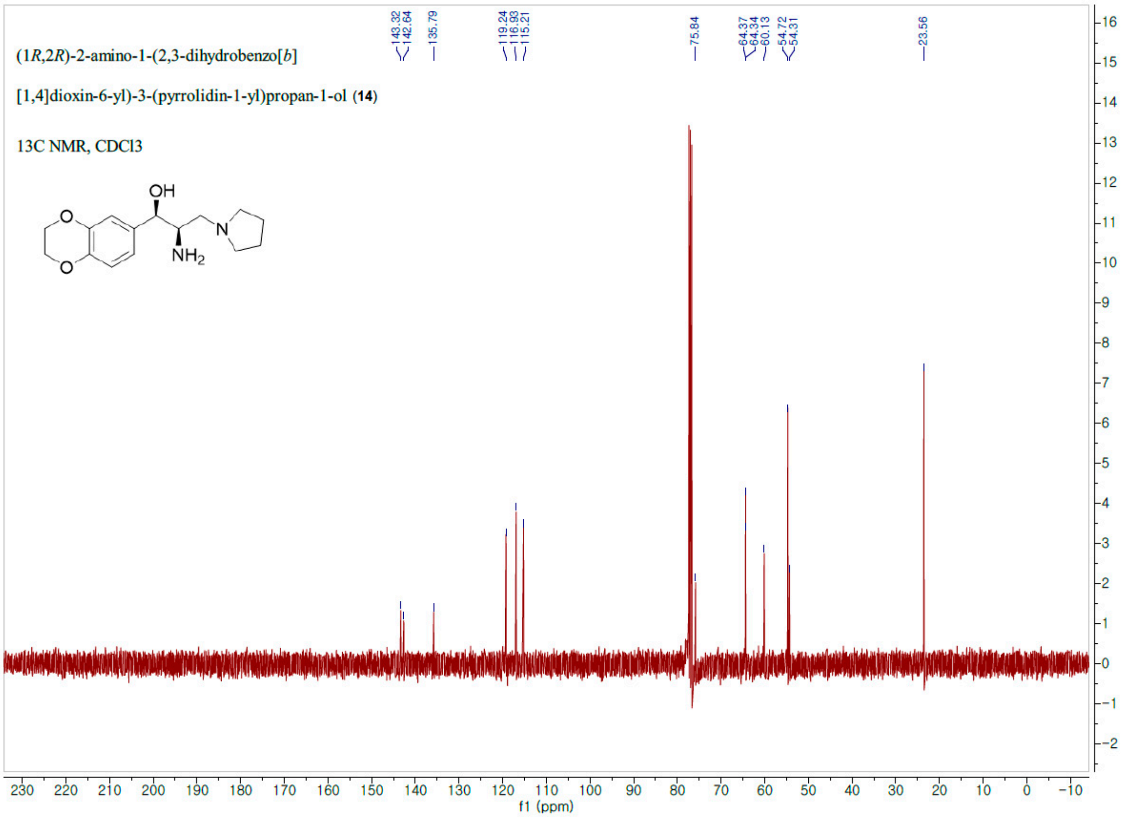

Figure S18. H and 13C NMR Spectra-S2-S11.

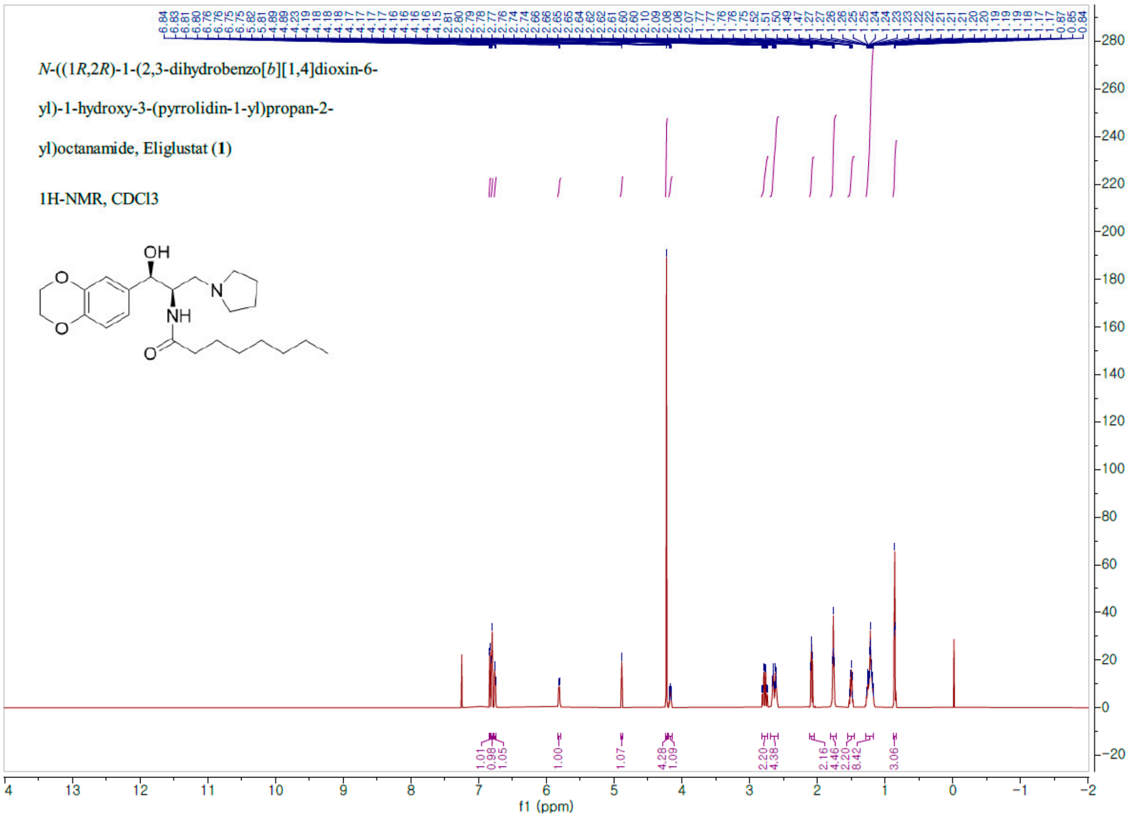

Figure S19. H and 13C NMR Spectra-S2-S11.

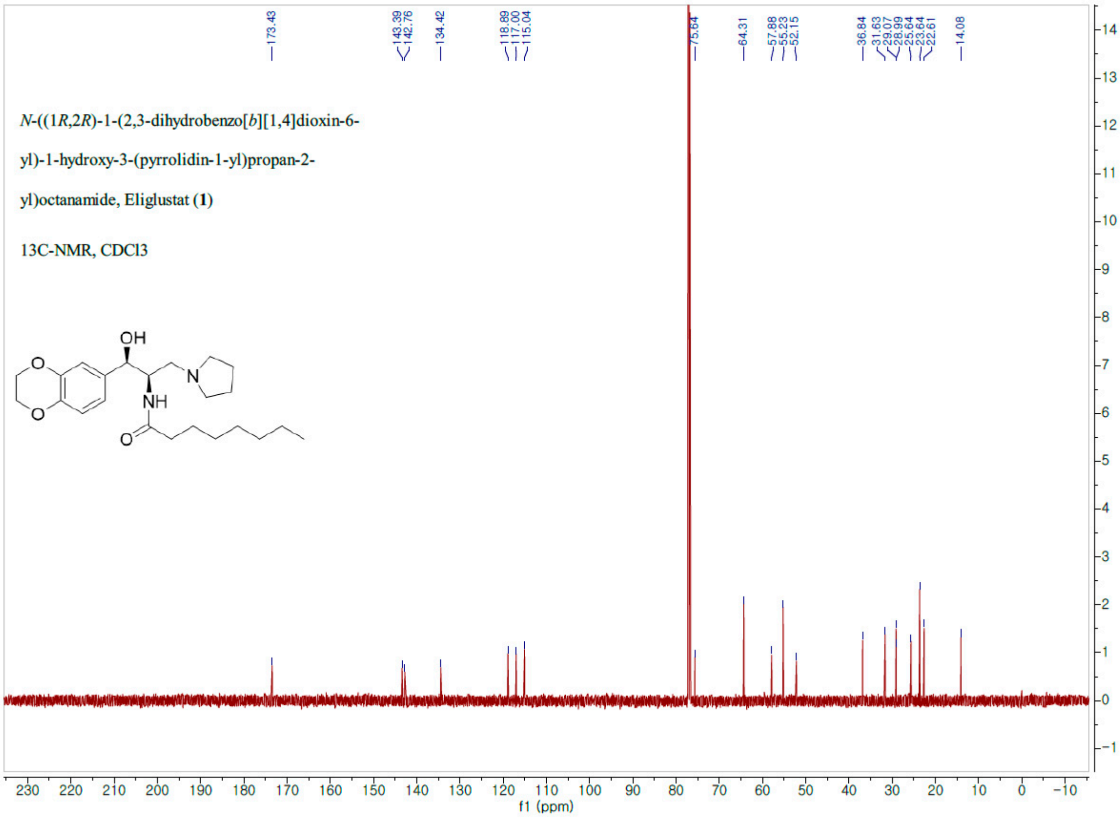

Figure S20. H and 13C NMR Spectra-S2-S11.
